# BET bromodomain-mediated interaction between ERG and BRD4 promotes prostate cancer cell invasion

**DOI:** 10.18632/oncotarget.9513

**Published:** 2016-05-20

**Authors:** Alexandra M. Blee, Shujun Liu, Liguo Wang, Haojie Huang

**Affiliations:** ^1^ Mayo Graduate School, Mayo Clinic College of Medicine, Mayo Clinic, Rochester, MN, 55905, USA; ^2^ Department of Biochemistry and Molecular Biology, Mayo Clinic College of Medicine, Rochester, MN, 55905, USA; ^3^ Division of Biomedical Statistics and Informatics, Mayo Clinic College of Medicine, Rochester, MN, 55905, USA; ^4^ Department of Urology, Mayo Clinic College of Medicine, Rochester, MN, 55905, USA; ^5^ Mayo Clinic Cancer Center, Mayo Clinic College of Medicine, Rochester, MN, 55905, USA; ^6^ The Hormel Institute, University of Minnesota, Austin, MN, 55912, USA

**Keywords:** ERG, BRD4, acetylation, bromodomain, cell invasion

## Abstract

Prostate cancer (PCa) that becomes resistant to hormone castration and next-generation androgen receptor (AR)-targeted therapies, called castration-resistant prostate cancer (CRPC), poses a significant clinical challenge. A better understanding of PCa progression and key molecular mechanisms could bring novel therapies to light. One potential therapeutic target is ERG, a transcription factor aberrantly up-regulated in PCa due to chromosomal rearrangements between androgen-regulated gene *TMPRSS2* and *ERG*. Here we show that the most common PCa-associated truncated ERG T1–E4 (ERGΔ39), encoded by fusion between *TMPRSS2* exon 1 and *ERG* exon 4, binds to bromodomain-1 (BD1) of bromodomain containing protein 4 (BRD4), a member of the bromodomain and extraterminal domain (BET) family. This interaction is partially abrogated by BET inhibitors JQ1 and iBET762. Meta-analysis of published ERG (T1–E4) and BRD4 chromatin immunoprecipitation-sequencing (ChIP-seq) data demonstrates overlap in a substantial portion of their binding sites. Gene expression profile analysis shows some ERG-BRD4 co-target genes are upregulated in CRPC compared to hormone-naïve counterparts. We provide further evidence that ERG-mediated invasion of PCa cells was significantly enhanced by an acetylation-mimicking mutation in ERG that augments the ERG-BRD4 interaction. Our findings reveal that PCa-associated ERG can interact and co-occupy with BRD4 in the genome, and suggest this druggable interaction is critical for ERG-mediated cell invasion and PCa progression.

## INTRODUCTION

Current therapies for localized PCa include surgery and radiation. However, for advanced or recurrent PCa, hormone castration therapy or treatment with androgen receptor (AR) inhibitors is a standard of treatment. These therapies inactivate the AR to halt transcription of androgen responsive target genes, but cancer cells often develop resistance. The resulting CRPC is difficult to eradicate, as these cancer cells mutate to restore AR signaling or progress independently of AR [1, 2]. Therefore, there exists a need for new therapeutics or combinations of therapies less prone to resistance. Interestingly, a frequent event in both primary and advanced PCa is the over-expression of ETS-related gene (ERG) transcription factor, which occurs in approximately half of all PCa, and much less frequently, other ETS family members such as ETV1, ETV4, and FLI1 [3–5]. In these cases, over-expression of N-terminally truncated ERG usually results from a chromosomal rearrangement. The rearrangement fuses protein-coding regions of *ERG* to the 5′ untranslated region (5′-UTR) of *TMPRSS2*, an androgen regulated gene [6]. ERG is increasingly recognized as a key component of PCa [7–10] and represents a promising target for PCa therapies, although regulation of ERG functions, such as by post-translational modifications, is poorly understood.

Several studies have previously identified promising strategies for cancer therapy that disrupt interactions between bromodomain proteins and oncogenic transcription factors [11–13]. BRD4 is of particular interest. BRD4 normally recognizes a diacetylated lysine motif on histone H4 to promote gene transcription by recruiting the CyclinT1/CDK9 complex [12, 14]. Interestingly, in basal-like breast cancer, an acetylatable histone H4 mimic motif within the oncogenic transcription factor TWIST can recruit BRD4 and activate transcription of *WNT5A*, which correlates with an aggressive cancer phenotype [15]. Inhibition of the TWIST-BRD4 interaction limits aggressive cancer behaviors such as cell invasion and stemness. In this study, we show that BRD4 interacts with T1-E4 ERG, and that ultimately this interaction promotes cell invasion. These findings present a novel mechanism of ERG-mediated PCa progression and highlight the use of BET inhibitors in PCa therapies.

## RESULTS

### Wild-type and PCa-associated T1-E4 ERG interact with BRD4

**Figure 1:**
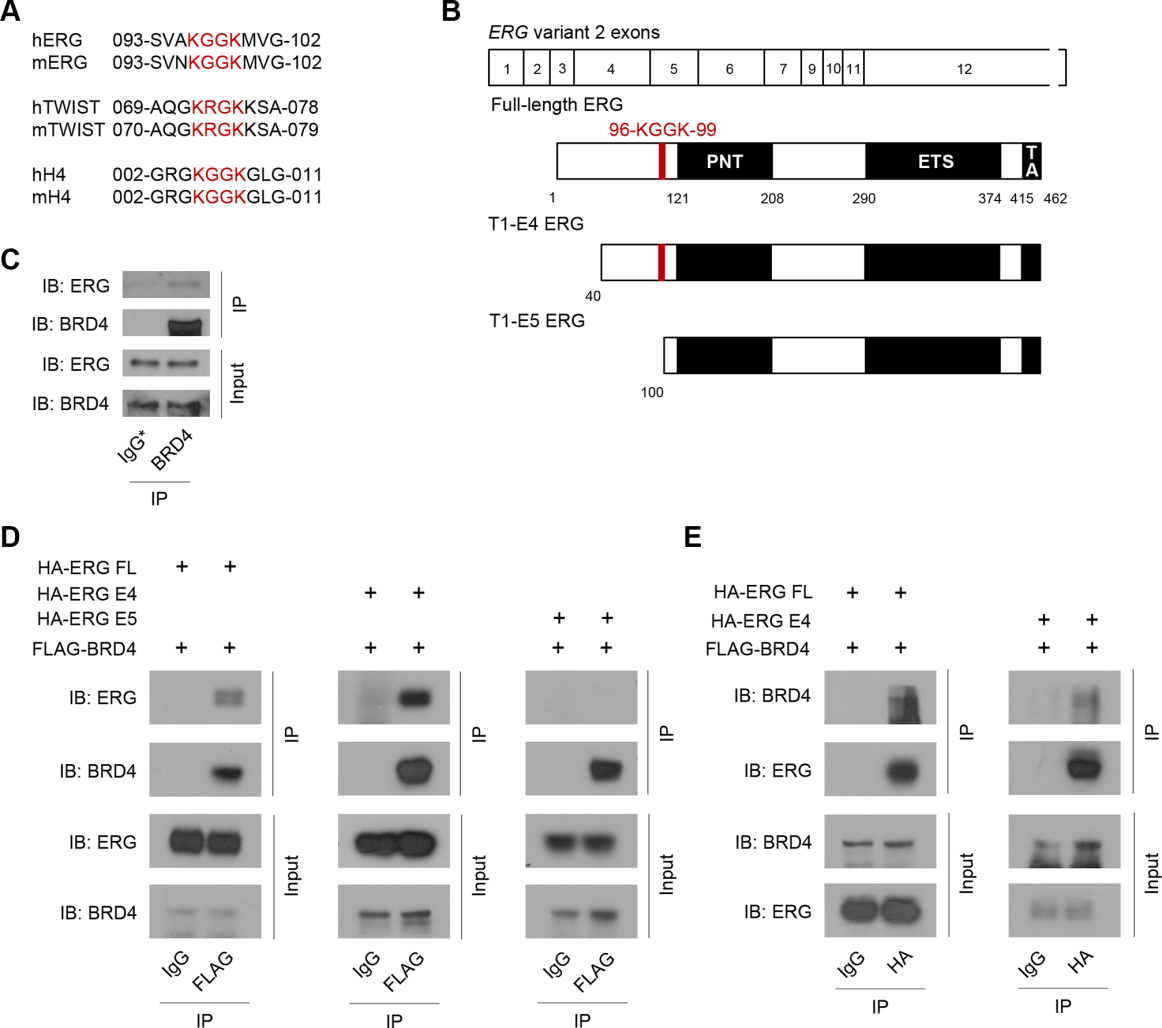
Wild-type and PCa-associated T1-E4 ERG interact with BRD4 (**A**) Protein sequence alignment between human (h) and mouse (m) ERG, TWIST, and histone H4 showing a conserved KGGK motif (red). (**B**) Schematic showing known domains of ERG and location of conserved KGGK motif (PNT domain, ETS DNA binding domain and TA transactivation domain). Exons for *ERG* mRNA variant 2 shown above. (**C**) Western blot showing BRD4 co-immunoprecipitation (co-IP) with endogenous BRD4 and T1-E4 ERG in VCaP cells. IgG* control co-IP performed with heat-inactivated BRD4 antibody (BRD4 antibody was heated to 95°C for 5 minutes prior to use). (**D**) Western blot showing FLAG co-immunoprecipitation with over-expressed FLAG-BRD4 and HA-ERG in HEK293T cells. IgG co-IP as a control. (**E**) Western blot showing reciprocal HA co-IP with over-expressing FLAG-BRD4 and HA-ERG in HEK293T cells. IgG co-IP as a control.

Examination of ERG protein sequence revealed the presence of a conserved BRD4-interacting motif ^96^KGGK^99^ (Figure 1A). In histone H4, lysine residues 5 and 8 of this motif are acetylated to recruit BRD4 and subsequently CyclinT1/CDK9 for transcription elongation at active promoters [12]. A similar motif has been identified in the transcription factor TWIST [15]. The presence of this motif in ERG suggests possible acetylation and interaction with BRD4 to initiate transcription of target genes in PCa. Interestingly, this motif is present in full-length ERG as well as the most commonly over-expressed PCa variant of ERG, T1-E4, but not another fairly commonly over-expressed variant, T1-E5 (Figure 1B) [16, 17]. The vertebral cancer of the prostate (VCaP) cell line was originally isolated from a cancer patient with PCa bone metastasis [18]. These cells harbor a *TMPRSS2*-*ERG* fusion and highly express T1-E4 truncated ERG. Co-immunoprecipitation of endogenous BRD4 and T1-E4 ERG in VCaP cells revealed interaction between these two proteins (Figure 1C). To confirm the interaction observed in VCaP cells, co-immunoprecipitation in HEK293T cells with ectopically expressed BRD4 and full-length, T1-E4, and T1-E5 ERG variants was performed. We found that BRD4 interacts with both full-length and T1-E4 ERG, but not T1-E5 ERG (Figure 1D). This result is consistent with the fact that T1-E5 ERG lacks the putative BRD4-binding motif ^96^KGGK^99^. Reciprocal co-immunoprecipitation with HA-tagged ERG confirmed the interactions between BRD4 and full-length or T1-E4 ERG (Figure 1E). These data indicate that wild-type and some PCa-associated variants of ERG bind to BRD4 and suggest that the ^96^KGGK^99^ motif may be important in mediating the interaction.

### Bromodomain-1 of BRD4 and ^96^KGGK^99^ of ERG are important for interaction

**Figure 2:**
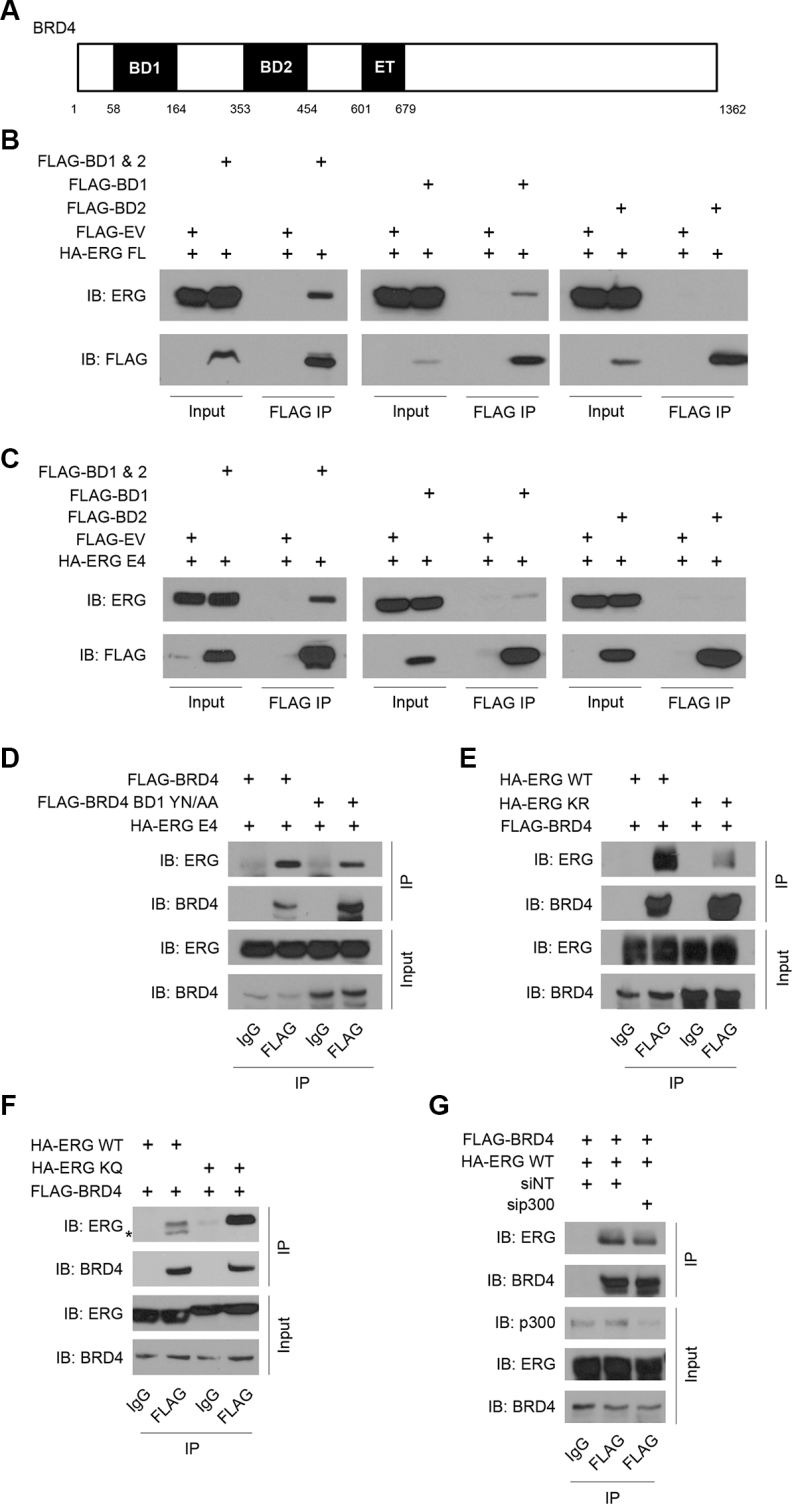
Bromodomain-1 of BRD4 and ^96^KGGK^99^ of ERG are important for interaction (**A**) Schematic showing known domains for BRD4, notably the two conserved bromodomains (BD1 and BD2) and the extraterminal (ET) domain. (**B**) Western blot showing FLAG co-IP with over-expressed FLAG-tagged full-length BRD4 or truncations and HA-tagged full-length ERG in HEK293T cells. FLAG co-IP in cells transfected with FLAG empty vector (EV) as a control. (**C**) Western blot showing FLAG co-IP with over-expressed FLAG-BRD4 or truncations and HA-ERG T1-E4 in HEK293T cells. FLAG co-IP in cells transfected with FLAG empty vector as a control. (**D**) Western blot showing FLAG co-IP with over-expressed FLAG-BRD4 or FLAG-BRD4 with mutated BD1 and HA-ERG T1-E4 in HEK293T cells. IgG co-IP as a control. (**E**) Western blot showing FLAG co-IP with over-expressed FLAG-BRD4 and HA-ERG full-length wild-type or acetylation-block mutant (KR) in HEK293T cells. IgG co-IP as a control. (**F**) Western blot showing FLAG co-IP with over-expressed FLAG-BRD4 and HA-ERG full-length wild-type or acetylation-mimic mutant (KQ) in HEK293T cells. IgG co-IP as a control. *Non-specific band. (**G**) Western blot showing FLAG co-IP with over-expressed FLAG-BRD4 and HA-ERG full-length wild-type with or without p300 knockdown in HEK293T cells. IgG co-IP as a control.

To further characterize the interaction between ERG and BRD4, we sought to identify the precise regions of ERG and BRD4 involved. BRD4 protein contains two bromodomains, bromodomain-1 (BD1) and -2 (BD2), located in the N-terminal half of the protein (Figure 2A). Each of these domains likely interacts with a pair of acetylated lysine residues [19]. A co-immunoprecipitation assay was performed with various BRD4 truncation mutants to identify the regions of BRD4 sufficient for the ERG-BRD4 interaction. These truncations included BD1 or BD2 alone or together. Co-immunoprecipitation with ectopically expressed full-length ERG and BRD4 truncation mutants revealed that full-length ERG interacts strongly with BD1 and BD2 together or slightly weaker with BD1 alone, but not with BD2 alone (Figure 2B). A similar result was observed after co-immunoprecipitation with ectopically expressed T1-E4 ERG and BRD4 truncation mutants (Figure 2C). Although relatively less ERG protein was observed after pull-down with BD1 than BD1 and 2 together, it appears that BD1 alone is sufficient for the interaction. One explanation for this result is that while BD1 alone is sufficient, the amino acids and protein structure immediately adjacent to BD1 are also important in mediating protein-protein interactions. To ensure that the BRD4 truncations did not drastically alter the bromodomain structures and functionality, we mutated highly conserved BD1 residues tyrosine 139 (Y139) and asparagine 140 (N140) in full-length BRD4 to alanine residues (YN/AA), as these residues are crucial for bromodomain activity [14]. Co-immunoprecipitation with ectopically expressed T1-E4 ERG and BRD4 YN/AA mutant revealed a decrease in interaction (Figure 2D). It is worth noting that these point mutations did not completely abolish binding, again suggesting that although BD1 alone is sufficient for binding, the conformation of BRD4 as a whole may also contribute to a more stable interaction. Taken together, these data suggest BD1 of BRD4 is sufficient for interaction with full-length and T1-E4 ERG, and that the acetylated lysine-binding function of BD1 is important.

Additionally, co-immunoprecipitation with full-length ERG protein where both lysine residues 96 and 99 within the ^96^KGGK^99^ motif were mutated to arginine (^96^RGGR^99^, or KR), a basic residue that cannot be acetylated, revealed decreased interaction between ERG and BRD4 (Figure 2E). Conversely, co-immunoprecipitation with full-length ERG protein where lysine residues 96 and 99 were mutated to glutamic acid (^96^QGGQ^99^, or KQ), a residue that mimics acetylated lysine [20], revealed increased interaction between ERG and BRD4 (Figure 2F). Lastly, because ERG is thought to be acetylated at lysine residues 96 and 99 by the histone acetyltransferase p300 [13], co-immunoprecipitation between ERG and BRD4 was performed in the presence of p300 knockdown (Figure 2G). As expected, knockdown of p300 diminished the ERG-BRD4 interaction. Based on these data, acetylation of the ^96^KGGK^99^ motif of ERG as well as BD1 of BRD4 are important for the interaction between these two proteins.

### BET inhibitors JQ1 and iBET762 diminish the ERG-BRD4 interaction

**Figure 3:**
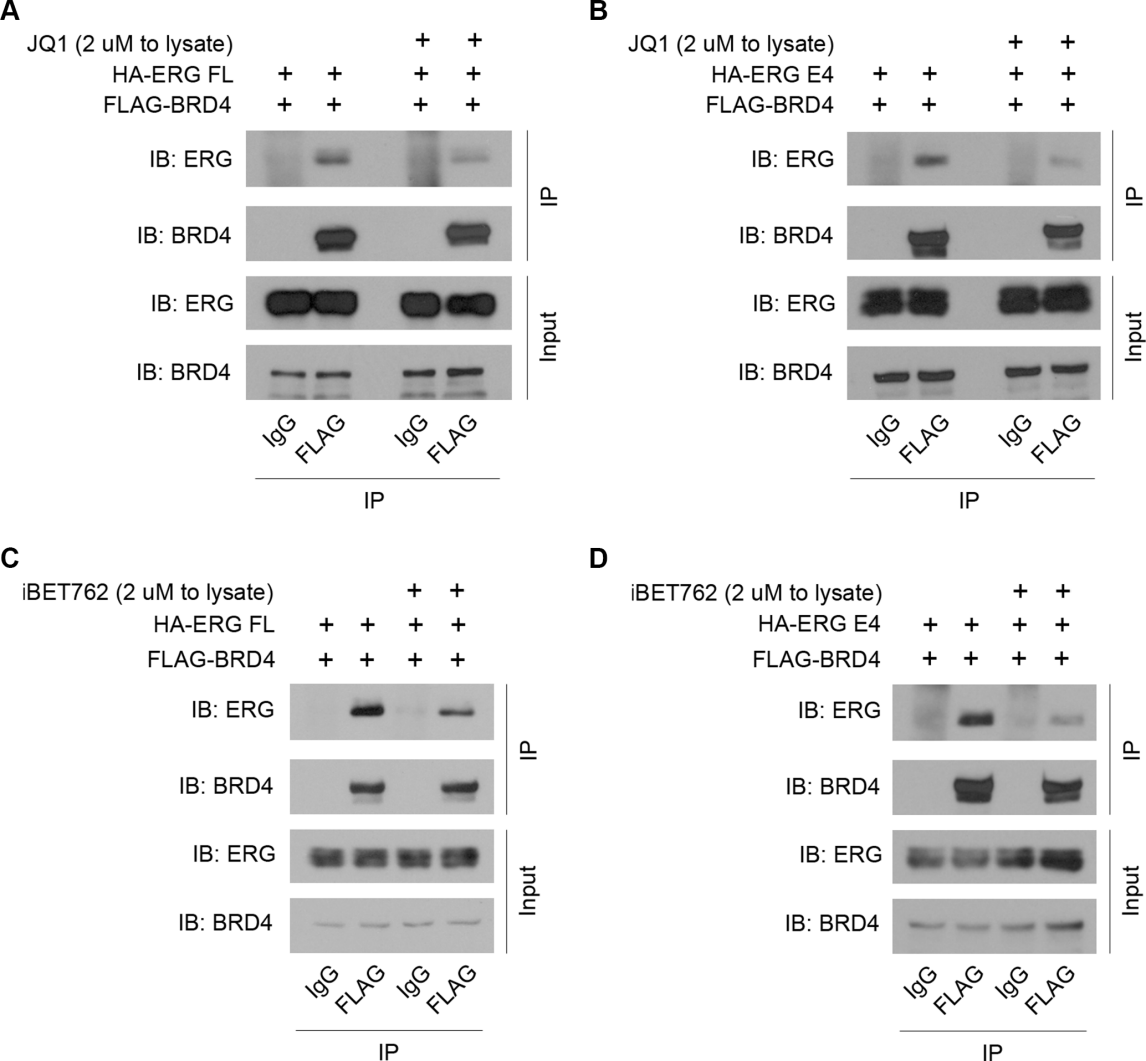
BET inhibitors JQ1 and iBET762 diminish the ERG-BRD4 interaction (**A**) Western blot showing FLAG co-IP with over-expressed FLAG-BRD4 and HA-ERG full-length in HEK293T cells, in the presence of 2 μM JQ1 or DMSO added directly to cell lysates. IgG co-IP as a control. (**B**) Western blot showing FLAG co-IP with over-expressed FLAG-BRD4 and HA-ERG T1-E4 in HEK293T cells, in the presence of 2 μM JQ1 or DMSO added directly to cell lysates. IgG co-IP as a control. (**C**) Similar set-up as in (A) but with 2 μM iBET762 or DMSO. (**D**) Similar set-up as in (B) but with 2 μM iBET762 or DMSO.

Identification of a specific therapy that targets ERG-mediated transcription could be extremely useful for clinical treatment of PCa. Because of the ERG-BRD4 interaction and its expected influence on gene transcription, BET inhibitors such as JQ1 [21] and iBET762 [22] are clear candidates. This class of inhibitors has already exhibited effective anti-cancer activities in preclinical models of many cancer types [23], including breast cancer [15], PCa [11, 24], acute myeloid leukemia [13], and show promise in other cancer types with a reliance on BET family proteins, such as squamous cell carcinoma [25]. Currently, two clinical trials for cancer therapy with iBET762 are ongoing (ClinicalTrials.gov identifiers: NCT01587703, NCT01943851). A clinical trial for another BET inhibitor, OTX015, a small molecule with a similar scaffold to JQ1, is also ongoing (ClinicalTrials.gov identifier: NCT02259114), demonstrating the great potential of such compounds for cancer therapies. Indeed, in HEK293T cells with ectopically expressed ERG and BRD4, addition of JQ1 to cell lysates before co-immunoprecipitation led to a noticeable decrease in the interaction between full-length ERG and BRD4 (Figure 3A), as well as between T1-E4 ERG and BRD4 (Figure 3B) after pull-down. iBET762 also partially disrupted the interaction between full-length ERG and BRD4 (Figure 3C), and between T1-E4 ERG and BRD4 (Figure 3D). Addition of these inhibitors directly to the cell lysate ensures that protein expression is not altered. Ten-fold higher concentrations of JQ1 did not have an increased inhibitory effect (Supplementary Figure S1). These data further support the notion that the bromodomain activity is important for BRD4 to bind to ERG.

### Endogenous ERG and BRD4 occupy the same subset of chromatin loci in VCaP PCa cells

**Figure 4:**
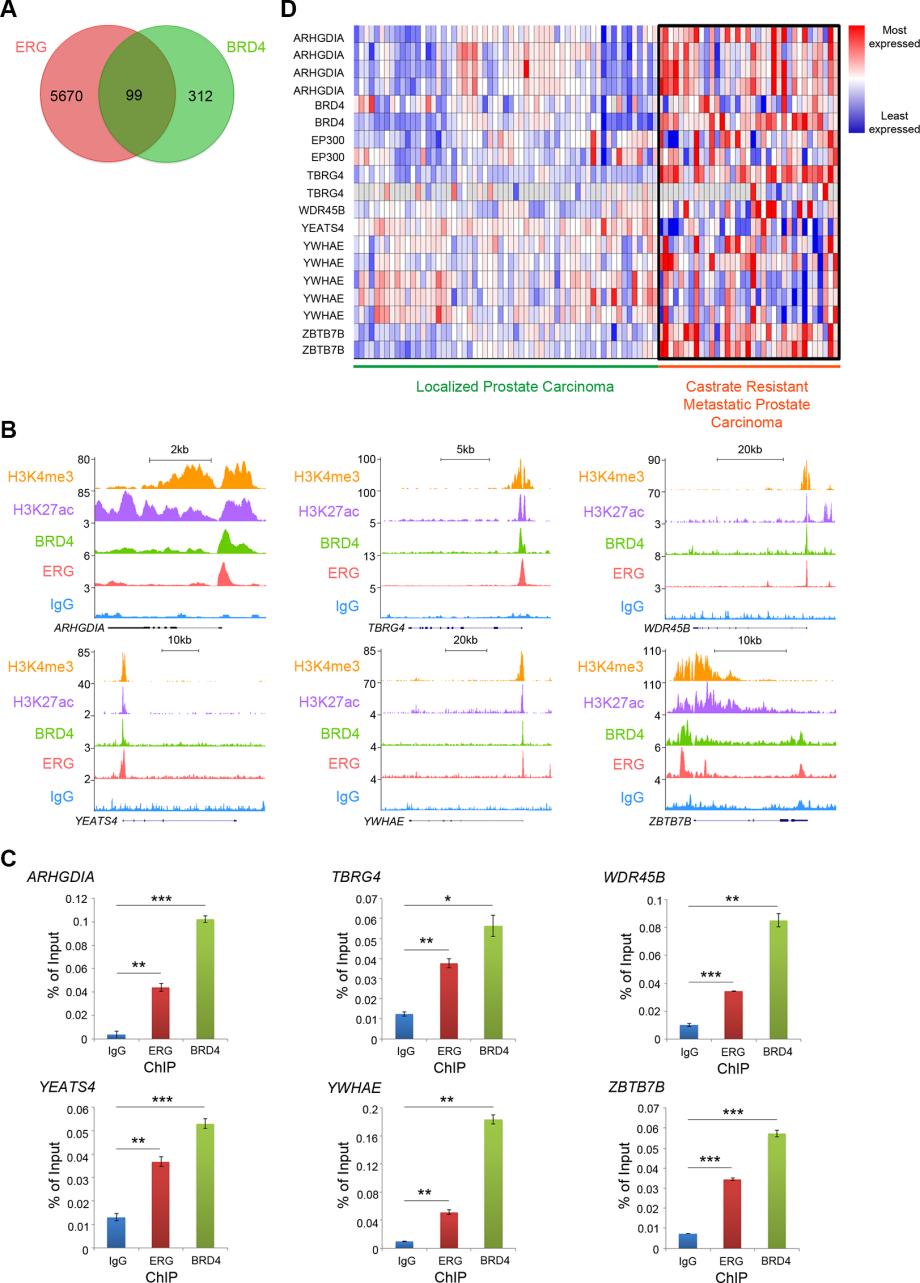
Endogenous ERG and BRD4 occupy the same subset of chromatin loci in VCaP PCa cells (**A**) Venn diagram showing the number of ERG or BRD4 ChIP-seq peaks at gene promoters and the number that overlapped. Overlapped binding sites were analyzed by the permutation test with *P* < 0.0001. VCaP ChIP-seq data for ERG and BRD4 were downloaded from NCBI Gene Expression Omnibus [10] with accession number GSE55064 [11]. (**B**) Previously published, publicly accessible ChIP-seq data from VCaP cells for BRD4 and ERG (T1-E4) IP, with IgG IP as a control. Six representative genes are shown. H3K4me3 and H3K27ac ChIP-seq data from LNCaP PCa cells shown to provide basic information about regions of gene promoter and active transcription. VCaP ChIP-seq data for ERG, BRD4, and IgG were downloaded from NCBI Gene Expression Omnibus [10] with accession number GSE55064 [11]. Signature profiles for H3K4me3 and H3K27ac in LNCaP cells were downloaded with accession numbers GSE43791 for H3K4me3 and GSE27823 for H3K27ac [26]. (**C**) ChIP-qPCR with ERG (T1–E4), BRD4, and IgG IP for each of the six genes to validate ERG and BRD4 binding in VCaP cells. *P < 0.05, **P < 0.01, ***P < 0.001. (**D**) Gene expression heat map from the Grasso PCa patient dataset [27], comparing expression of genes from 59 localized and 35 CRPC patient samples. Compiled with the ONCOMINE cancer microarray database [54].

To determine the common chromatin binding sites of endogenous T1-E4 ERG and BRD4, we performed meta-analysis of publicly available ChIP-seq data of T1-E4 ERG and BRD4 in VCaP cells. VCaP ChIP-seq data for ERG, BRD4, and IgG were reported previously [11] (GSE55064). Signature profiles for H3K4me3 (GSE43791) and H3K27ac (GSE27823) in LNCaP cells were also reported previously [26]. After analysis of two independent datasets for both ERG and BRD4 to identify well-conserved binding sites, the permutation test was used to determine the number of gene promoters bound by both ERG and BRD4. There was significant overlap between ERG and BRD4 peaks at the promoter regions of 99 genes (as evidenced by enrichment of the authentic promoter histone mark, histone H3 lysine 4 trimethylation, H3K4me3), suggesting these two proteins have the potential to work in concert to regulate expression of their co-target genes (Figure 4A). As shown in Figure 4B, six candidate genes, including *ARHGDIA*, *TBRG4*, *WDR45B*, *YEATS4*, *YWHAE* (14-3-3ε), and *ZBTB7B*, exhibit significant co-occupancy of BRD4 and ERG. These genes were chosen for further studies because they have strong links to cancer progression and cell proliferation. Binding of both endogenous ERG and BRD4 at these gene promoters was validated in VCaP cells using ChIP followed by quantitative PCR (ChIP-qPCR) (Figure 4C). Analysis of the previously published prostate patient sample dataset [27] revealed that the majority of these genes, including *ARHGDIA*, *TBRG4*, *WDR45B*, and *ZBTB7B*, were up-regulated in advanced PCa compared to primary PCa (Figure 4D). Interestingly, *EP300* and *BRD4* were also up-regulated in advanced PCa compared to primary PCa (Figure 4D). This suggests that although *TMPRSS2-ERG* fusion only occurs in approximately 50% of both primary and advanced tumors [3, 4], overexpression of other transcriptional regulators such as p300 and BRD4 may be a critical factor that drives up-regulation of ERG-BRD4 co-target genes specifically in advanced tumors.

Many of these genes also play certain roles in cancer progression. For example, the 14-3-3 protein family member 14-3-3ε (YWHAE) is linked with high-grade and metastatic endometrial stromal sarcomas [28–30]. 14-3-3ε can also induce epithelial-to-mesenchymal transition (EMT) in hepatocellular carcinoma (HCC) [31]. YEATS domain containing 4 (YEATS4) protein represses p53 during the normal cell cycle [32] and is implicated in gliomas [33], colorectal cancer [34], and non-small cell lung cancer [35]. High expression of transforming growth factor beta regulator 4 (TBRG4) correlates with poor outcome for multiple myeloma patients [36]. Of particular interest was zinc finger and BTB domain containing protein 7B (ZBTB7B) transcription factor. ZBTB7B is a known oncogene in lymphoma and is thought to promote cell transformation and oncogenesis by suppressing apoptosis in cells with Ras and Myc activation [37, 38]. These data suggest that the ERG-BRD4 interaction could regulate gene expression in PCa cells and impact cancer cell behavior such as invasion.

### Acetylation-mimic ERG increases prostatic cell invasion

**Figure 5:**
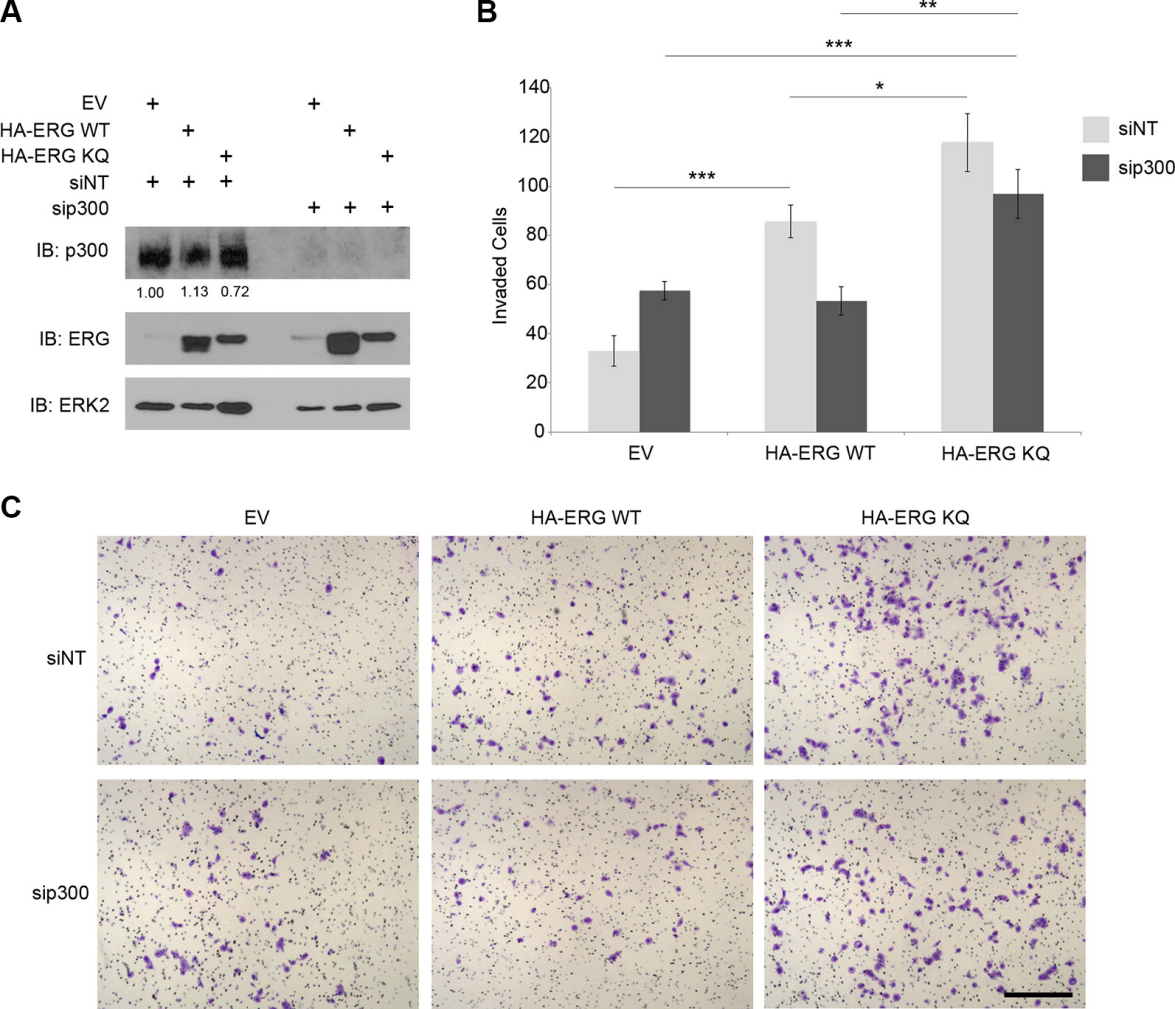
Acetylation-mimic ERG increases prostatic cell invasion (**A**) Western blot for ERG and p300 with over-expressed HA-ERG full-length wild-type (WT) or HA-ERG full-length acetylation-mimic (KQ), and p300 siRNA knockdown in BPH-1 cells. As controls, cells were transfected with non-targeting siRNA (siNT), and empty vector (EV). ERK2 as a loading control. p300 protein levels were normalized to ERK2 first and then to the value for cells transfected with EV and siNT. (**B**) Number of BPH-1 cells that invaded through basement membrane and matrix with over-expressed empty vector, HA-ERG wild-type, HA-ERG acetylation-mimic (KQ), with or without p300 siRNA. *P < 0.05, **P < 0.01, ***P < 0.001. (**C**) Representative images from BPH-1 cell invasion assay. Scale bar, 200 μm.

ERG over-expression in PCa is linked with increased cell invasion and metastasis [6, 9, 39]. Interestingly, RNAi-mediated inhibition of ERG in VCaP cells abrogates aggressive cancer cell behavior [9, 40]. As mentioned above, several of the genes identified by ChIP-seq and validated by ChIP-qPCR have the potential to regulate cell invasion and cancer progression. To determine whether ERG-BRD4 interaction affects cell behavior, immortalized but non-transformed benign prostatic hyperplasia (BPH-1) cells were used for a transwell invasion assay. These cells were transfected with full-length wild-type or acetylation-mimic ERG, as well as siRNA specific for p300 (Figure 5A). As expected, BPH-1 cells over-expressing full-length ERG invaded through basement membrane and matrix approximately twice as frequently as control BPH-1 cells without ERG (Figure 5B and 5C). In addition, BPH-1 cells over-expressing the acetylation-mimic ERG exhibited a significantly higher rate of invasion than those over-expressing wild-type ERG (Figure 5B and 5C). This data is consistent with the finding that the acetylation-mimic ERG interacts more with BRD4 than wild-type ERG (Figure 2F), and suggests the ERG-BRD4 interaction is functionally important in cancer.

p300 was recently identified as an acetyltransferase that acetylates ERG to allow binding with BRD4 [13], and interestingly, p300 is up-regulated in PCa [41]. As a result, knockdown of p300 is expected to disrupt the effect of wild-type ERG on cell invasion. However, loss of p300 would not be expected to affect the acetylation-mimic motif in the KQ mutant ERG. Indeed, RNAi-mediated knockdown of p300 abrogated the effect of wild-type ERG on cell invasion, but not of acetylation-mimic ERG (Figure 5B and 5C). It is worth noting that knockdown of p300 alone increased invasion in BPH1 cells without ERG overexpression (Figure 5B and 5C). The molecular mechanism underlying such a cell context-dependent effect is unknown at present and warrants further investigation. Nevertheless, these results suggest that the interaction between acetylated ERG and BRD4 plays an important role in prostatic cell invasion.

### ERG-BRD4 co-targets and canonical ERG targets cooperate in prostatic cells

**Figure 6:**
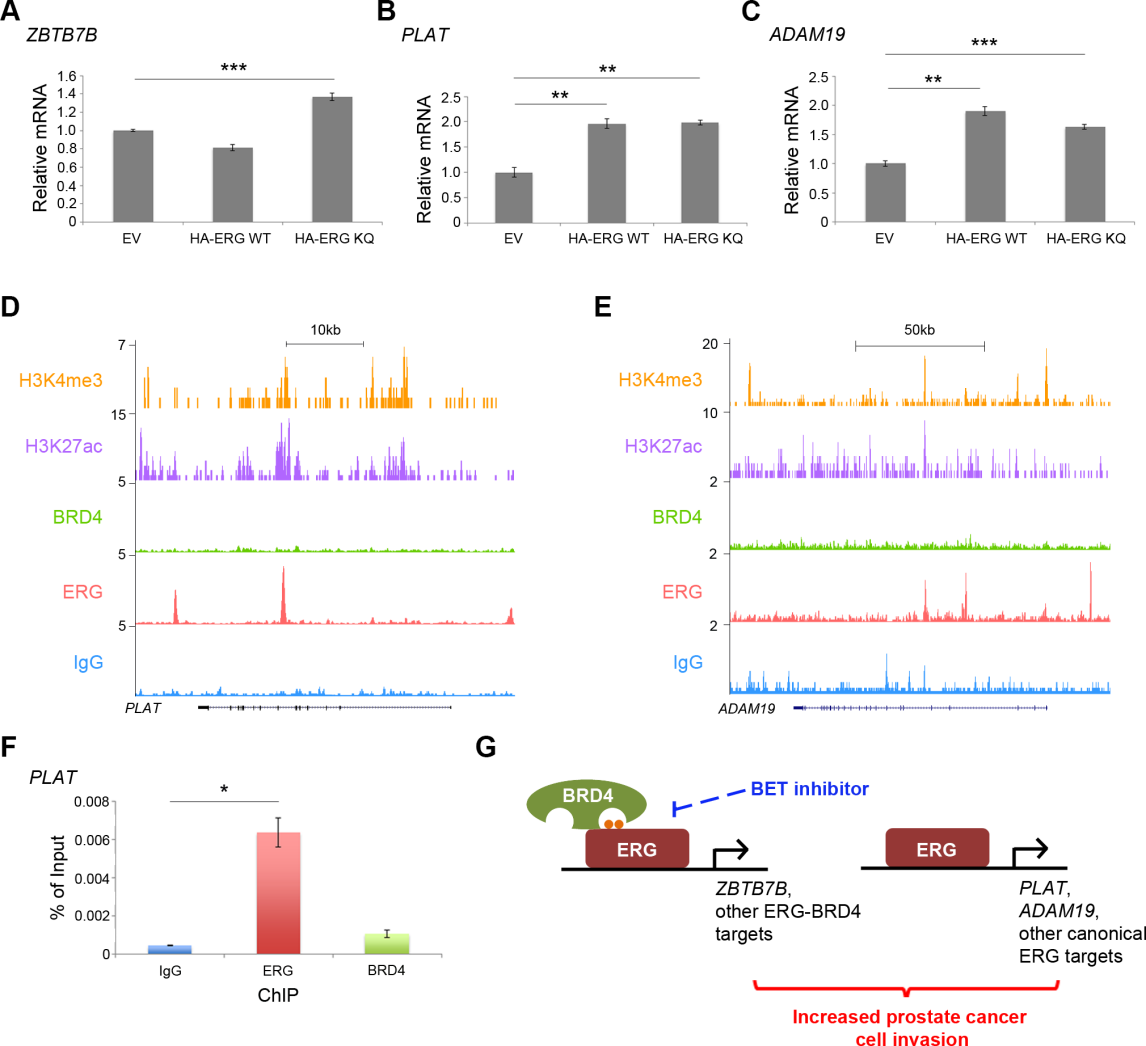
ERG-BRD4 co-targets and canonical ERG targets cooperate in prostatic cells (**A**) RT-qPCR for *ZBTB7B* in BPH-1 cells with over-expressed empty vector (EV), HA-ERG full-length wild-type (WT) or HA-ERG acetylation-mimic mutant (KQ). ***P < 0.001. (**B**) RT-qPCR for *PLAT* in BPH-1 cells with over-expressed EV, HA-ERG WT, or HA-ERG KQ. **P < 0.01. (**C**) RT-qPCR for *ADAM19* in BPH-1 cells with over-expressed EV, HA-ERG WT, or HA-ERG KQ. **P < 0.01, ***P < 0.001. (**D** and **E**) Published BRD4 and ERG ChIP-seq data from VCaP cells [11]. Published H3K4me3 and H3K27ac ChIP-seq data from LNCaP cells [26] provide evidence indicating the regions of active transcription. (**F**) ERG and BRD4 ChIP-qPCR show the binding of ERG, but not BRD4 at the *PLAT* locus. *P < 0.05. (**G**) A hypothetical model. Acetylation-dependent interaction between ERG and BRD4 promotes transcription of co-target genes, and may contribute to increased cell invasion in combination with transcription of canonical ERG target genes. This ERG-BRD4 interaction can be targeted with BET inhibitors. Small orange dots represent acetylation sites on ERG protein.

To investigate the mechanism by which ERG-BRD4 interaction promotes invasion, the BPH-1 cells over-expressing wild-type or acetylation-mimic ERG (Figure 5A) were used for further functional analysis. We found that BPH-1 cells transfected with acetylation-mimic ERG expressed higher levels of *ZBTB7B* mRNA, a BRD4-ERG cotarget gene (Figure 4), compared to cells expressing control vector or wild-type ERG (Figure 6A). This suggests ERG interaction with BRD4 under optimal conditions (e.g. when ERG is acetylated or has the acetylation mimic KQ motif) is important for expression of *ZBTB7B*. Due to the lack of reports that ZBTB7B alone can directly regulate cell invasion, canonical ERG target genes such as matrix metalloproteases and members of the plasminogen activator pathway were also analyzed [9, 10]. Tissue plasminogen activator (*PLAT*) and a disintegrin and metalloproteinase 19 (*ADAM19*) mRNA levels were similarly increased in BPH-1 cells over-expressing both wild-type ERG and acetylation-mimic ERG compared to control cells (Figure 6B and 6C). This result suggests that expression of canonical ERG targets *PLAT* and *ADAM19* is regulated by ERG independently of ERG acetylation and interaction with BRD4, unlike expression of the ERG-BRD4 co-target gene *ZBTB7B*. Accordingly, as demonstrated by ERG and BRD4 ChIP-seq in VCaP cells at *PLAT* and *ADAM19* gene loci (Figure 6D and 6E) and ChIP-qPCR at the *PLAT* locus, BRD4 did not co-localize with ERG (Figure 6F). Together, these data suggest that expression of ERG-BRD4 co-target genes such as *ZBTB7B* in combination with expression of canonical ERG target genes such as *PLAT* and *ADAM19* may lead to a significant increase in aggressive cell behavior such as invasion (Figure 6G).

## DISCUSSION

As the second leading cause of cancer-related death in American men, CRPC poses a challenge in clinic [42]. Clearly, a better understanding of the molecular mechanisms of PCa progression and resistance are necessary to better treat this lethal disease. While it is well accepted that ERG is frequently over-expressed in PCa [3–5], and that ERG can mediate PCa progression and cell invasion [5, 6, 43, 44], the mechanisms underlying ERG-mediated transcription and cell invasion remain largely unclear. In particular, post-translational modification and regulation of ERG, such as interaction with co-activators and -repressors, is not well understood.

In this study, we demonstrate that ERG and BRD4 interact in a manner dependent on bromodomain activity and ERG acetylation to affect prostatic cell behavior. Importantly, the most common PCa-associated N-terminally truncated ERG, T1-E4, interacts with BRD4, providing a possible mechanism for ERG-mediated transcription in PCa. This interaction can be disrupted by BET inhibitors, a class of small molecules currently in clinical trials for cancer therapy. Additionally, we show that these two proteins co-localize at a subset of gene promoters in PCa cells. In support of these observations, we demonstrate that an acetylation-mimic mutant of ERG can increase prostatic cell invasion to a greater extent than wild-type ERG, particularly in the presence of p300 knockdown. Therefore, our findings demonstrate a possible mechanism of ERG-mediated transcription that regulates aggressive PCa behavior, and suggest that disruption of the ERG-BRD4 interaction may be a useful addition to current therapies.

While we are investigating the interaction between BRD4 with wild-type and PCa-associated variants and its role in PCa cell invasion, it has been shown recently that wild-type ERG is acetylated by p300 and interacts with BRD4 to initiate gene transcription in acute myeloid leukemia (AML) [13]. In this case, inhibition of bromodomain proteins also shows promise in ameliorating the cancer phenotype. It is interesting to note that in PCa, ERG is frequently N-terminally truncated at varying lengths due to the nature of the chromosomal rearrangements that lead to ERG over-expression [7, 45, 46]. Both ERG in AML and PCa-associated ERG such as T1-E4 contain the ^96^KGGK^99^ motif important for acetylation and interacting with BRD4. However, another common PCa-associated form of ERG, T1-E5, lacks this motif and does not interact with BRD4, suggesting that there may be additional mechanisms for ERG-mediated transcription in PCa beyond interaction with BRD4. This finding stresses the importance of individualized treatment for PCa, as any one tumor may be heterogeneous and express different N-terminally truncated variants of ERG that may respond differently to therapies.

In summary, we demonstrate that ERG and BRD4 interact with each other in PCa and that their interaction is important for PCa cell invasion. Due to high homology between the bromodomains of many BET family members, it is possible that multiple BET proteins such as BRD2 or BRD3 can recognize ERG and mediate similar outcomes [23]. Nevertheless, our findings suggest that blocking the ERG-BRD4 interaction through BET inhibitors is a promising therapy for PCa that expresses the relevant ERG variants. While BET inhibitors are already known to target AR-BRD4 interaction to indirectly decrease *TMPRSS2-ERG* expression through inhibition of the *TMPRSS2* promoter [11], this study further demonstrates another mechanism by which BET inhibitors may abrogate PCa progression. ERG variant stability can be regulated in an AR-independent manner, through evasion of SPOP-mediated proteasome degradation [47, 48]. As a result, specifically disrupting the ERG-BRD4 interaction with BET inhibitors may serve an equally important, yet disparate purpose to prevent PCa progression, in addition to disrupting the AR-BRD4 interaction and AR-mediated expression of *TMPRSS2-ERG* fusions. In combination with other therapies such as protein acetyltransferase inhibitors including I-CBP112 and C646 [49, 50] or therapies targeting other key PCa mutations, disruption of the ERG-BRD4 interaction may prove highly effective for a subset of advanced PCa.

## MATERIALS AND METHODS

### Plasmids, antibodies, and chemicals

The mammalian pCMV expression vectors for HA-tagged full-length wild-type ERG, HA-tagged E4 ERG (Δ39), and HA-tagged E5 ERG (Δ99) were described previously [47]. HA-tagged ERG K96Q/K99Q and K96R/K99R mutants were generated from HA-tagged full-length ERG in the pCMV vector with the KOD-Plus Mutagenesis kit (Toyobo). Mammalian pCMV-Tag2B expression vectors for FLAG-tagged full-length BRD4, FLAG-tagged BD1, FLAG-tagged BD2, and FLAG-tagged BD1 and 2 were kindly provided by Dr. Binhua P. Zhou [15]. pCMV-Tag2B empty vector was kindly provided by Dr. Martin Fernandez-Zapico (Mayo Clinic). FLAG-tagged full-length BRD4 Y139A/N140A was generated from FLAG-tagged full-length BRD4 in the pCMV-Tag2B vector with the KOD-Plus Mutagenesis kit (Toyobo). Antibodies used were: anti-BRD4 (E2A7X, Cell Signaling Technology); anti-ERG (CM421A, Biocare Medical); anti-ERG (sc353 and sc354, Santa Cruz Biotechnology), anti-ERK2 (sc1647, Santa Cruz Biotechnology), anti-p300 (sc585, Santa Cruz Biotechnology); anti-FLAG (M2, Sigma-Aldrich); anti-HA 1.1 (Covance); anti-light chain specific rabbit IgG secondary antibody (211-032-171, Jackson Immuno Research Laboratories); ECL anti-rabbit (or anti-mouse, GE Healthcare UK Limited); IgG horseradish peroxidase linked whole antibody; mouse IgG (Vector Laboratories, Inc.). JQ1 was kindly provided by Dr. James Bradner and purchased from Sigma-Aldrich. iBET762 was purchased from MedchemExpress.

### Cell lines, cell culture and transfection

The cell lines HEK293T and VCaP were purchased from ATCC (Manassas, VA). BPH-1 cells were kindly provided by Dr. Donald Tindall (Mayo Clinic). HEK293T cells were cultured in Dulbecco's modified Eagle's medium (Corning cellgro) supplemented with 10% FBS (Thermo Fisher Scientific, Cat# 10437028). VCaP cells were cultured in Dulbecco's modified Eagle's medium (Corning cellgro) supplemented with 13% FBS (Thermo Fisher Scientific, Cat# 10437028). BPH-1 cells were cultured in RPMI 1640 medium (Corning cellgro) supplemented with 10% FBS (Thermo Fisher Scientific, Cat# 10437028). Transfections were performed using Lipofectatmine2000 (Thermo Fisher Scientific) or by electroporation using an Electro Square Porator ECM 830 (BTX) with Mirus Ingenio solution, following manufacturer's instructions. Approximately 75–90% transfection efficiencies were routinely achieved, verified by expression of GFP co-transfected with the plasmid(s) of interest.

### RNA interference

Cells were transfected with siRNA following manufacturer's instructions, by electroporation as described above. Non-specific control siRNA was purchased from RIBOBIO (siN05815122147) and siRNA for human p300 was custom-designed and purchased from Thermo Fisher Scientific Dharmacon (5′-GGACUACCCUAUCAAGUAAAU-3′).

### Co-immunoprecipitation and Western blotting

Cells were harvested and lysed using sonication with diagenode Bioruptor UCD-200 in cell lysis buffer (50 mM Tris-HCl, pH 7.5, 150 mM NaCl, 1% Nonidet P-40, 0.5% sodium deoxycholate and 1% protease inhibitor cocktails, Sigma-Aldrich). Cell lysates were centrifuged and the supernatant was incubated with indicated antibodies and Protein G Plus/Protein A Agarose beads (Calbiochem) or FLAG M2-conjugated agarose beads (Sigma Aldrich) at 4°C overnight. The beads were washed six times with cell lysis buffer and the precipitated proteins were further analyzed. For western blotting, equal amounts of protein (80–100 micrograms) from cell lysates were denatured in sample buffer (Thermo Fisher Scientific), subjected to SDS-polyacrylamide gel electrophoresis (Bio-Rad) and transferred to nitrocellulose membranes (Thermo Fisher Scientific). The membranes were blocked in 1× TBST with 5% milk, immunoblotted with indicated primary antibodies at 4°C overnight, followed by incubation at room temperature for 1 hour with horseradish peroxidase-conjugated secondary antibodies. Blots were visualized by SuperSignal West Pico Luminol Enhancer Solution (Thermo Fisher Scientific). BET inhibitors (JQ1 and iBET762) or DMSO as control were added directly to cell lysate (2 μM) during incubation with beads.

### Mining of published ERG, BRD4, H3K4me3, and H3K27ac ChIP-seq data

VCaP ChIP-seq data for ERG, BRD4, and IgG were reported previously [11] and downloaded from NCBI Gene Expression Omnibus [10] with accession number GSE55064. Signature profiles for H3K4me3 and H3K27ac in LNCaP cells were reported previously [26] with accession numbers GSE43791 for H3K4me3 and GSE27823 for H3K27ac. All datasets were reprocessed. In brief, raw reads were aligned to human reference genome (hg19/GRCh37) using Bowtie2 (v2.1.0) [51], and unique mapped reads were further used for peak calling by MACS2 (v2.0.10) [52] with *q*-value < 0.05. The peak-gene association analysis was performed by GREAT (http://bejerano.stanford.edu/great/public/html/).

### Chromatin immunoprecipitation coupled quantitative polymerase chain reaction (ChIP-qPCR)

The ChIP assay was performed as described previously [53]. VCaP cells were cultured in DMEM medium with 13% serum. Cells were crosslinked with 1% formaldehyde for 10 minutes at room temperature. Crosslinking was quenched by adding 2.5 M glycine to a final concentration of 125 mM. Cells were scraped from the dish and rinsed twice with 1× PBS. Cells were lysed for 10 minutes at 4°C in lysis buffer 1 (50 mM HEPES, pH 7.5, 140 mM NaCl, 1 mM EDTA, 10% glycerol, 0.5% NP-40, 0.25% TritonX-100) followed by 10 minutes at room temperature in lysis buffer 2 (10 mM Tris-HCl, pH 8.0, 200 mM NaCl, 1 mM EDTA). Nuclei were pelleted and sonicated to lyse nuclei and shear crosslinked DNA into smaller fragments in lysis buffer 3 (10 mM Tris-HCl, pH 8.0, 100 mM NaCl, 1 mM EDTA, 0.1% sodium deoxycholate, 0.5% NP-40). Cell lysis was centrifuged and the supernatant was incubated overnight at 4°C with 35 μl Protein G Plus/Protein A Agarose beads (Calbiochem) pre-blocked in 0.5% BSA in 1X PBS and pre-incubated with 10 μg indicated antibodies. Beads were washed 7 times with IP buffer (50 mM HEPES, pH 7.5, 500 mM LiCl, 1 mM EDTA, 1% NP-40, 0.7% sodium deoxycholate) and once with TE buffer containing 50 mM NaCl. Precipitated DNA was eluted by heating to 65°C for 15 minutes in elution buffer (50 mM Tris-HCl, pH 8.0, 10 μM EDTA, 1% SDS). 0.2 μg/ml RNase A was added to digest cellular RNA and 0.2 μg/ml proteinase K was added to digest cellular protein. DNA was extracted with a PureLink Quick PCR purification kit (Thermo Fisher Scientific) and subjected to real-time PCR amplification using primers specific for the promoters of genes analyzed. The data for chromatin occupation was expressed as a ratio of the cycle threshold for the immunoprecipitated chromatin DNA versus the cycle threshold for the input. Primers: *ARHGDIA* forward 5′-GTCACCTCTGTAAGCCAGGG-3′ and reverse 5′-GC CCGTGTTTAACGAGAACT-3′; *TBRG4* forward 5′-AGT ACGCCATCCTCATACGG-3′ and reverse 5′-TCCCCAG TCTCCACTCACTC-3′; *WDR45B* forward 5′-TTACACG GCAGGAGGTTCAT-3′ and reverse 5′-CTCCATAGGCT CCCTGGTG-3′; *YEATS4* forward 5′-GTTTCAGGTTGGA GAGCGAG-3′ and reverse 5′-CACCGCTTCACCAATAA CCT-3′; *YWHAE* forward 5′-AACTCACCGTCGTATC GCTC-3′ and reverse 5′-GAGTCGGAGACACTATCCG C-3′; *ZBTB7B* forward 5′-ATCCTTCACCCGCTACC TTT-3′ and reverse 5′-GCTTGACATGAATTCGCTGA-3′; *PLAT* forward 5′-TGTCATCACAGGGTCCTGAA-3′ and reverse 5′-TAAAGCAGGGGGAGGAAGTT-3′.

### Oncomine gene expression analysis of grasso prostate cancer dataset

Expression of *ARHGDIA*, *TBRG4*, *WDR45B*, *YEATS4*, *YWHAE*, *ZBTB7B, BRD4* and *EP300* in the Grasso et al. prostate cancer dataset [27] was analyzed using Oncomine (https://www.oncomine.com).

### Reverse transcription quantitative polymerase chain reaction (RT-qPCR)

Total RNA was isolated from cells and cDNA was synthesized using a GoScript kit (Promega). Two-step real-time polymerase chain reaction (PCR) was performed using the SYBR Green Mix (Bio-Rad) and C1000 Touch Thermal Cycler, CFX96 Real-Time System (Bio-Rad) according to manufacturer's instructions. Both forward and reverse primers were used at a final concentration of 500 nM. Expression of the *GAPDH* gene in each sample was used as an internal control. Primers: *GAPDH* forward 5′-ACCCACTCCTCCACCTTTGAC-3′ and reverse 5′-TG TTGCTGTAGCCAAATTCGTT-3′; *ZBTB7B* forward 5′-G ATCCTGACCTGATGGCCTA-3′ and reverse 5′-TGTGG ATCTTCAGCTTGTCG-3′; *PLAT* forward 5′-CACTGG GCCTGGGCAAACATA-3′ and reverse 5′-CACGTCAGC CTGCGGTTCTTC-3′; *ADAM19* forward 5′-GCCTATGCC CCCTGAGAGTG-3′ and reverse 5′-GCTTGAGTTGGCC TAGTTTGTTGTTC-3′.

### Cell invasion

Cell invasion was monitored by crystal violet staining using the Corning matrigel invasion chamber assay according to manufacturer's instructions (Corning). Briefly, BPH-1 cells were transfected with indicated plasmids and cultured in RPMI medium with 10% serum for 12 hours. Cells were then plated in Corning matrigel invasion chambers in 24-well plates at a density of 3 × 10^4^ cells per chamber and cultured in serum-free RPMI medium, with RPMI medium containing 10% FBS outside the chambers. After 24 hours, cells were fixed in methanol for 15 minutes followed by staining with 1 mg/ml crystal violet in 10% ethanol for 30 minutes. After rinsing with water 3 times, the membranes of the chambers were mounted and covered on slides and observed using a light microscope. Eight fields of view each from three independent replicates were recorded and analyzed.

### Statistical analysis

Experiments were carried out with three or more replicates unless noted otherwise. Statistical analyses were performed by Student's *t*-test for most studies. Values with *P* < 0.05 are considered statistically significant.

## SUPPLEMENTARY MATERIALS FIGURE


