# Supplementary material for: BET bromodomain-mediated interaction between ERG and BRD4 promotes prostate cancer cell invasion
Source: Oncotarget. 2016 May 20;7(25):38319–32. doi: 10.18632/oncotarget.9513 (PMC5122392; doi:10.18632/oncotarget.9513)
Supplement: Supplementary file 1 [file oncotarget-07-38319-s001.pdf]

BET bromodomain-mediated interaction between ERG and BRD4 promotes prostate cancer cell invasion

Supplementary Materials

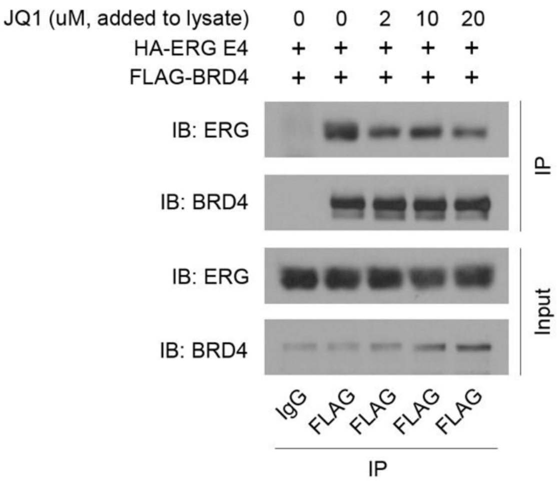

Supplementary Figure S1: JQ1 diminishes the ERG-BRD4 interaction. Western blot showing anti-FLAG antibody-mediated co-IP of over-expressed FLAG-BRD4 and HA-ERG T1-E4 in HEK293T cells. Increasing concentrations of JQ1 or DMSO were added directly to the cell lysate. IgG co-IP as a control.
